# Camera traps reveal extensive anthropogenic impacts inside protected areas in Bangladesh

**DOI:** 10.1371/journal.pone.0347792

**Published:** 2026-04-28

**Authors:** Raf Ana Rabbi Shawon, Md. Matiur Rahman, Md Mehedi Iqbal, Junji Moribe

**Affiliations:** 1 Laboratory of Wildlife Resources, Gifu University, Gifu, Japan; 2 Department of Medicine, Sylhet Agricultural University, Sylhet, Bangladesh; 3 Institute for East China Sea Research, Nagasaki University, Nagasaki, Japan; Salim Ali Centre for Ornithology and Natural History, INDIA

## Abstract

Camera traps serve as an essential tool for monitoring the wildlife habitat while simultaneously detecting and assessing various anthropogenic intrusions within protected areas (PAs). This study aims to monitor anthropogenic intrusions within the PAs of Satchari National Park (SNP) of Sylhet in Bangladesh. The study was conducted from May 2024 to April 2025 using camera traps that were systematically installed in 19 different stations inside the SNP. The study detected a total of 8,042 counts of videos that provided a large number of various anthropogenic intrusions inside SNP. Importantly, the study significantly revealed several anthropogenic intrusions including hunting, poaching, wood collection, tourism, and livestock grazing inside SNP. Notably, our results demonstrated that hunters and poachers in SNP carried firearms and traditional tools, including bows and arrows, confirming active hunting practices and diverse hunting methods. Moreover, the temporal co-efficient analysis indicated that anthropogenic activities were associated with shifts in wildlife activity patterns and temporal dynamics. The current study indicates that such disturbances could potentially impact and disrupt the ecological balance and pose a threat to the long-term persistence of wildlife within PAs of SNP. The present study also suggests the importance of maintaining anthropogenic intrusions at minimal levels in SNP and strengthening eco-tourism management to reduce conflicts between wildlife conservation and human recreation. To the best of our knowledge, this is the first camera trap monitoring to comprehensively evaluate anthropogenic activities in SNP of Bangladesh.

## Introduction

Forests play a vital role for preserving the global biodiversity, ensuring ecological balance, and supporting diverse wild animal species and their habitats that serving as critical markers of the planet’s general health [1,2]. Forests also provide livelihoods and cultural importance for indigenous and local communities that frequently rely on forest resources for food, traditional customs, and economic endeavors [3]. The International Union for Conservation of Nature (IUCN) defines a protected areas (PAs) as a definite geographical space managed, through legal or other effective means, for the long-term conservation of nature, and associated ecosystem services [4,5]. These PAs play a vital role for the biodiversity conservation, facilitate the preservation of degraded ecosystems, and provide useful frameworks that regulate human activities to align conservation goal with sustainable socio-economic development [4,6]. Currently, more than hundred thousand PAs worldwide cover over 12% of the Earth’s terrestrial surface and represent the most significant strongholds for biodiversity and landscape conservation [7].

The existing PAs are now encountering risks due to natural phenomena and various anthropogenic intrusions inside and outside of it [8,9]. Anthropogenic intrusions disturbances the functioning of natural ecosystems that can significantly affect wild animal species diversity, transform ecological architecture, change competitive interactions among wild animal species, and influence the availability and distribution of resources [10]. Previous studies reported that various anthropogenic activities including logging, mining, energy development, agricultural expansion, water conservancy projects, tourism, and urbanization impact the biodiversity of PAs [11,12]. Other studies have reported the effects of key anthropogenic activities such as tourism, hiking, camping, birdwatching, off-road driving, and mountain biking on wildlife, demonstrating impacts on wild animal behavior, habitat use, and overall biodiversity [13,14]. Such perturbations can induce modifications in wild animal species activity patterns, shifts in eating behaviors, and potential displacement from essential habitats. Over the time, these responses may reduce habitat suitability, disrupt species interactions, and ultimately compromise wild animal populations viability and ecosystem stability.

Previous study has reported that illegal activities, particularly hunting and land clearing, severely impact forest ecosystems by eliminating wildlife that play crucial roles in seed dispersal globally [15]. Such disruptions alter forest structure by favoring small-seeded tree species over large-seeded ones, ultimately reducing biodiversity and contributing to the degradation of forest landscapes [16]. The recent study reported that forest logging both in unprotected areas and illegally within PAs has increased the anthropogenic intrusions, thereby intensifying hunting pressure for commercial trade and for subsistence needs of logging camps in Southeast Asia [17]. The hunters may sometimes apply modern various instruments such as firearms and flashlights, unselective methods including snares and traps that require minimal effort, leading to shifts in faunal species population [18]. As a consequence, wildlife exploitation has substantially reduced the distribution and abundance of many wild animal species in Southeast Asia, resulting in severe local-scale biodiversity loss [19]. Moreover, the local community-driven hunting, poaching, and encroachment in PAs has also led to a decrease in the abundance and distribution of wild animal species in Bangladesh [20].

Satchari National Park (SNP) is one of the notable PAs that located in the Sylhet division of Bangladesh, distinguished for its unique biodiversity, tropical evergreen to semi-evergreen forests, and various wild animal species [21]. The previous study reported that local communities engage in activities within SNP, including illegal logging, livestock grazing inside the forest, uncontrolled wood collection, and agricultural encroachment, which contribute to ecosystem degradation and reduce the availability of resources for indigenous wildlife [22]. Moreover, SNP has experienced a steady increase in tourism activities over the decades, introducing additional disturbance to wildlife and natural habitats [23]. Moreover, anthropogenic intrusions are occurring throughout the year inside the SNP; however, these issues have not yet been scientifically documented using camera trap methods, largely due to unavoidable constraints. Notably, these studies relied on social survey–based evidence rather than systematic camera trap monitoring; therefore, the actual spatiotemporal patterns and real-time dynamics of anthropogenic activities and wildlife responses were not were not systematically quantified or documented. Camera trapping has become a powerful, non-invasive tool of modern research in wildlife, enabling long-term monitoring of species presence and other activity patterns while providing a discreet and effective means of observing targets [24,25]. Therefore, monitoring shifts in wild animal activity patterns offers valuable insights into ecological dynamics and helps assess how anthropogenic disturbances influence wildlife existence and conservation outcomes [26]. Our current study adopts a hypothesis-driven approach structured around the following research questions such as: (i) is there definite evidence of the presence of different types of anthropogenic disturbances within SNP? (ii) to what extent do human activity patterns temporally overlap with wildlife activity? (iii) is increased human presence associated with reduced wildlife habitat or shifts in diel activity patterns? and (iv) do different categories of anthropogenic disturbance differentially influence or impact wildlife activity patterns? Therefore, this study aims to systematically monitor and identify the real-time scenario of anthropogenic disturbances and evaluate their spatiotemporal dynamics on wildlife activity patterns within SNP of Bangladesh.

## Materials and methods

### Study area

**Fig 1:**
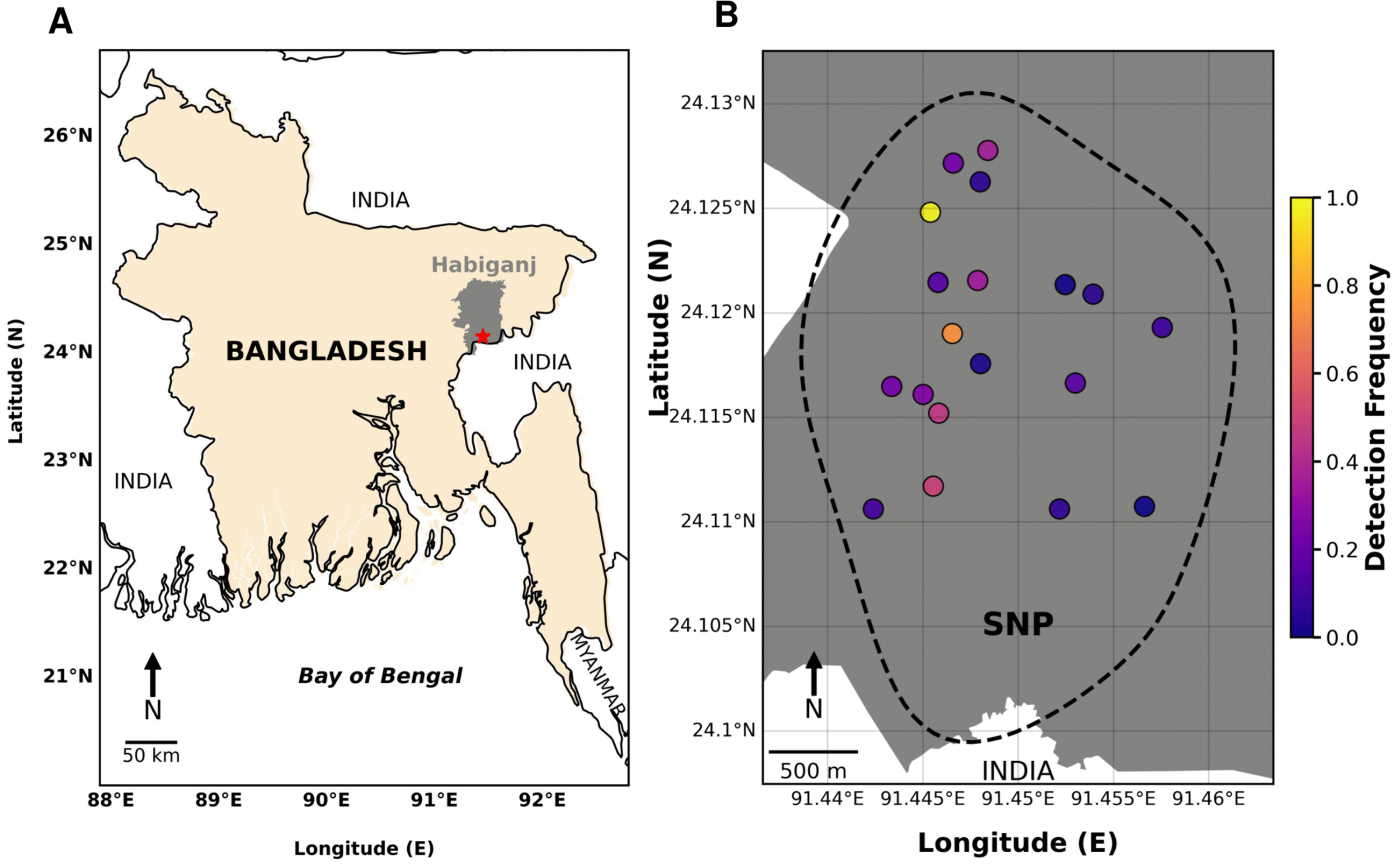
Study area of camera trap. A map of Bangladesh indicating the precise study area location of SNP within the Habiganj district of Sylhet division, highlighted in green box **(A)**. A comprehensive map of SNP illustrating the geographical arrangement of camera trap locations, indicated by red diamond marks **(B)**.

Bangladesh is home to a wide range of natural landscapes, notably semi-evergreen to evergreen forest ecosystems distributed across several regions including the Sylhet division [27]. The camera trap survey was carried out in SNP of Sylhet, located at 24°07′12″ N 91°27′03″ E, encompassing approximately of 2.43 km^2^ of mixed-evergreen forest (Fig 1). This evergreen forest plays a vital role as a biodiversity hotspot, hosting a variety of flora and fauna, semi-evergreen to evergreen vegetation, complex topography, and high biodiversity [28]. Geographically, SNP of Sylhet is defined by undulating hills and elevated mounds within a tropical monsoon climate, receiving an average annual rainfall of approximately 5,173 mm. The forest landscape is bounded with seasonal, sand-bottomed streams that contribute to habitat heterogeneity and overall ecological richness [29]. The SNP is also surrounded by human settlements and extensive tea gardens. A national highway runs along the northern boundary of the Raghunandan Hill Reserved Forest (RHRF), another protected area adjacent to SNP that effectively dividing the SNP landscape. Ecological connectivity between SNP and the RHRF is preserved through five small bridges that act as wildlife underpasses, facilitating animal movement along stream corridors. The close interaction between natural features and anthropogenic elements underscores the importance of SNP as a key site for conservation research and biodiversity management.

### Camera trap

The camera trap was conducted over a one-year period ranging from May 2024 to April 2025 in SNP of Sylhet, using a total of nine digital wildlife cameras (TC06, China) across 19 sampling stations. The camera stations were systematically selected and installed along frequently used wildlife pathways including feeding and resting areas, valley corridors, and stream margins, to maximize the detection probability of the wild animals and anthropogenic intrusions according to the previous reports with some modifications [30,31]. Briefly, each camera was securely mounted on the tree trunks at a height of approximately 30–60 cm above ground level using heavy chains to ensure stability. To reduce the risk of damage, human interference, or theft, all camera traps were housed in reinforced iron protective cases. We didn’t provide any bait or attractants, ensuring unbiased documentation of natural wildlife activity. To enhance video quality, surrounding undergrowth, including foliage and small branches within the camera detection zone, was carefully cleared to avoid false triggers and visual obstruction. The motion-sensor setting was configured to capture three consecutive still photographs per trigger, followed immediately by a 10 seconds video clip to document the wild animal movement and anthropogenic intrusions. All cameras were operated continuously for 24 hours per day with a minimum inter-trigger interval of 10 seconds and infrared flashes were set to low-glow mode to minimize disturbance. Before the full operation, each camera was field-tested to verify sensor responsiveness, images and videos clarity, correct time, date stamping, and appropriate detection angles through test walks in front of the cameras. To ensure continuous camera trapping operation throughout the 12-months survey period, field inspections were performed at bi-weekly to check and replace memory cards and batteries as needed. Additionally, selected cameras were repositioned at intervals of 1–1.5 months to improve spatial coverage and capture a broader range of targets.

### Data analysis

Data obtained from the camera trap videos were methodically organized using Flexible Renamer (ver. 8.4) and NeoFileInfolist (ver. 1.4.1.0) software. Notably, we identified the wild animal species along with the anthropogenic intrusions according to the previously published studies [32–34]. For further validation, we consulted experts including field wildlife guides from SNP, ecologists, and specialist academicians to verify the camera-trap data. All collected camera trap videos were manually inspected to distinguish genuine wildlife detections and anthropogenic intrusions from false triggers. The study carefully documented instances of anthropogenic intrusions including hunting, poaching, wood collection, tourism, livestock activity to assess their impact on wildlife and habitat within SNP. Our field teams carefully documented comprehensive notes for every instance of human actions observed to guarantee precise recording. Anthropogenic intrusions were categorized into specific categories such as hunting, poaching, wood collection, and tourism for various assessment. The categories were combined with wild animal observed video data from camera traps to examine potential links between anthropogenic intrusions and alterations in wildlife distribution, activity levels, and habitat utilization. Additionally, during video capture, some targets may trigger the camera multiple times while remaining in the detection zone, resulting in multiple videos of the same individual within a single event. To minimize the impact of repeated captures on Relative Abundance Index (RAI) values, duplicate videos were excluded. Consecutive videos of the same targets were taken within a 30 minutes interval that considered a single-event occurrence [35]. The RAI was estimated as (number of independent pictures/ total monitoring days) × 100 according to the previous study [36]. The RAI formula is in the following below:


$$RAI\;=A_{i}/N\times 100$$

where *A*_*i*_ represents the number of independent videos of wild animal species in category *i*, and *N* denotes the number of valid camera-trap days [35]. The processed data were subsequently analyzed and visualized using the Python packages ‘numpy’ [37], ‘pandas’ [38], ‘seaborn’ [39] and ‘Matplotlib’ [40]. Seasonal fluctuations were analyzed by categorizing monthly data into three distinct intervals: the pre-monsoon hot season (March–June), the monsoon wet season (July–October), and the dry winter season (November–February). To complement the correlation analysis, the study quantified temporal overlap between wildlife species and anthropogenic activities using kernel density estimation of detection times across the 24-hour cycle module in Python [41]. The overlap was showed as the coefficient of overlap (Δ), ranging from 0 (no overlap) to 1 (complete overlap). We estimated 95% confidence intervals using bootstrap resampling. We also used the Python packages ‘Cartopy’ (v0.22) and ‘Matplotlib’ [40] to generate the study map, utilizing shapefile data from the Global Administrative Areas (GADM) database [42].

### Ethical statement

Our research does not involve invasive or experimental procedures conducted on live animals. This study adheres strictly to observational or non-invasive methodologies, and no animal was harmed or subjected to experimental manipulation during the research. According to the ethical guidelines and regulations established by Gifu University, research of this nature does not fall within the scope of projects requiring formal ethical review or approval by the institutional animal care and use committee (IACUC) or equivalent bodies.

## Results

**Fig 2:**
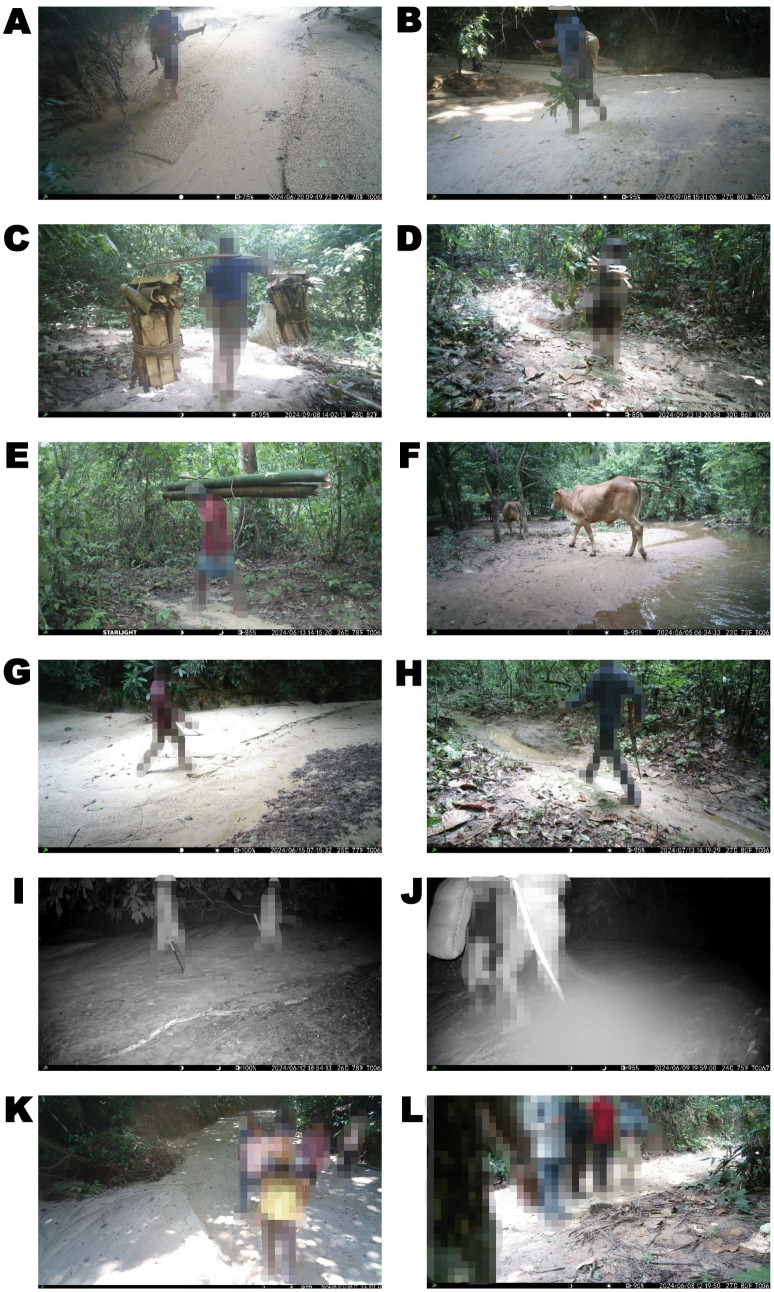
Various captured images of anthropogenic activities within SNP by camera traps. (A-E) wood collection, (F) livestock activity, (G-I) hunting, (J) poaching, (K-L) tourism.

Over a 12-months period, our camera trap recorded a total of 8,042 events by camera trapping in SNP of Sylhet that focused on wild animal species and various anthropogenic activities representing the majority of detections (Fig 2; Table 1). Importantly, wood collection was the most frequently detected human activity, with 442 independent events resulting in a relative abundance index (RAI) of 11.21 ± 3.47. On the other hand, tourism was also prominent with an RAI of 7.64 ± 2.61. On the other hand, RAI values of poaching was 7.30 ± 2.12 and hunting was 3.20 ± 0.85. Additionally, movements of forest patrolling (RAI = 0.96 ± 0.14), the presence of livestock activity (RAI = 2.80 ± 1.17), and research activity (RAI = 1.29 ± 0.22) were also detected by camera trapping within the SNP (Table 1). Furthermore, 1,834 counts of video data were obtained from the camera traps that have categorized as blanks without any objects, presumably triggered by environmental influences such as wind or the movement of plants. We excluded the research activity category from subsequent activity pattern and other analyses because it was not classified as an anthropogenic activity. We retained forest patrolling in the dataset because it represents a management and conservation activity that is important for our study.

**Table 1:** Identification of anthropogenic intrusions using camera trap surveys in SNP of Sylhet, Bangladesh.

Name	Count	PI (%)	RA (%)	RAI overall	Mean RAI	SE RAI	RAI (Mean ± SE)
Wood collection	442	5.49	5.49	10.03	11.21	3.47	11.21 ± 3.47
Tourism	248	3.08	3.08	5.63	7.64	2.61	7.64 ± 2.61
Hunting	104	1.29	1.29	2.36	3.20	0.85	3.20 ± 0.85
Poaching	237	2.95	2.95	5.38	7.30	2.12	7.30 ± 2.12
Forest patrolling	20	0.25	0.25	0.45	0.96	0.14	0.96 ± 0.14
Livestock activity	65	0.81	0.81	1.47	2.80	1.17	2.80 ± 1.17
Research activity^a^	33	0.41	0.41	0.75	1.29	0.22	1.29 ± 0.22

RA, relative abundance; IP, PI, proportion of independent photographs (%); RAI, relative abundance index; SE, standard error. a) Research activity was excluded from the other analyses.

**Fig 3:**
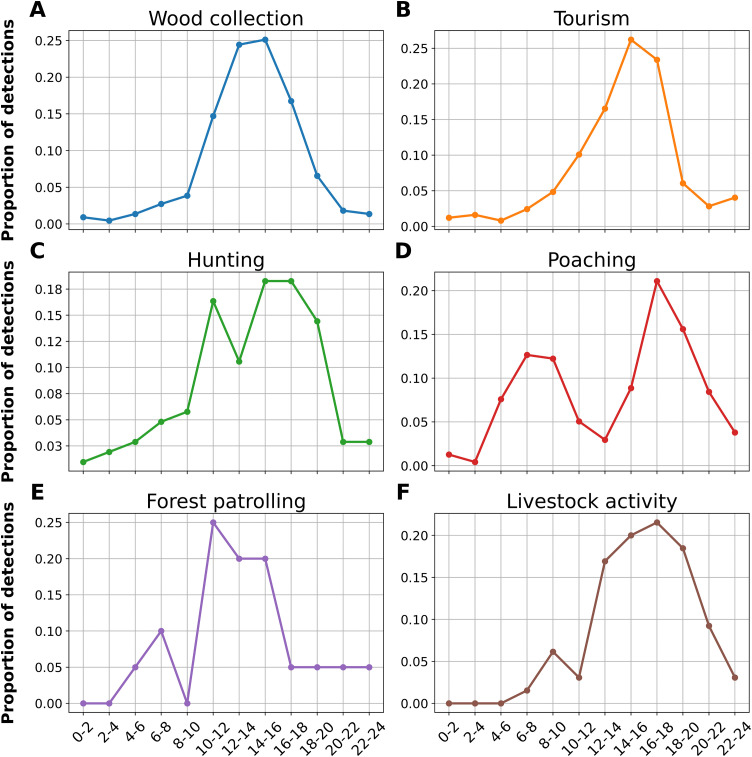
Diel activity patterns of various anthropogenic activities within SNP by camera traps. The frequency plotted against two-hour time intervals throughout a 24-hour period. The x-axis represents time in two-hour intervals, while the y-axis indicates the frequency of detections, reflecting the proportion of activity occurrences relative to the total observations. Shaded areas around the lines represent standard errors, indicating the variability in activity frequency across time intervals.

The diel activity pattern of various anthropogenic intrusions were demonstrated in Fig 3. The majority of wood collection activity took place in the middle of the day, with a notable increase beginning at 10:00 and reaching its highest point between 12:00 and 14:00 before decreasing once more in the late afternoon (Fig 3A). The time distribution of tourism is similar as well, with little activity in the early hours and a noticeable an increase between 12:00 and 14:00, followed by a slow decrease (Fig 3B). This midday peak coincides with accessible hours and standard travel timetables. On the other hand, hunting activity exhibits a more dispersed pattern, with two slight peaks: one in the morning (around 8:00–10:00) and another in the late afternoon (around 14:00–16:00) (Fig 3C). The unlawful and frequently covert poaching activity has a clearly bimodal pattern, with higher activity during the early morning hours (6:00–8:00) and a stronger peak in the late afternoon (16:00–18:00), presumably to evade detection by forest staff or other visitors (Fig 3D). The activity of forest patrolling shows two distinct peaks, one at midday (12:00–14:00) and another early in the morning (6:00–8:00), which correspond to regular patrols or management tasks that are usually carried out during the day (Fig 3E). Activities associated with livestock show a distinct peak in the late afternoon (16:00–18:00), with some activity noted in the morning (8:00–10:00) (Fig 3F).

**Fig 4:**
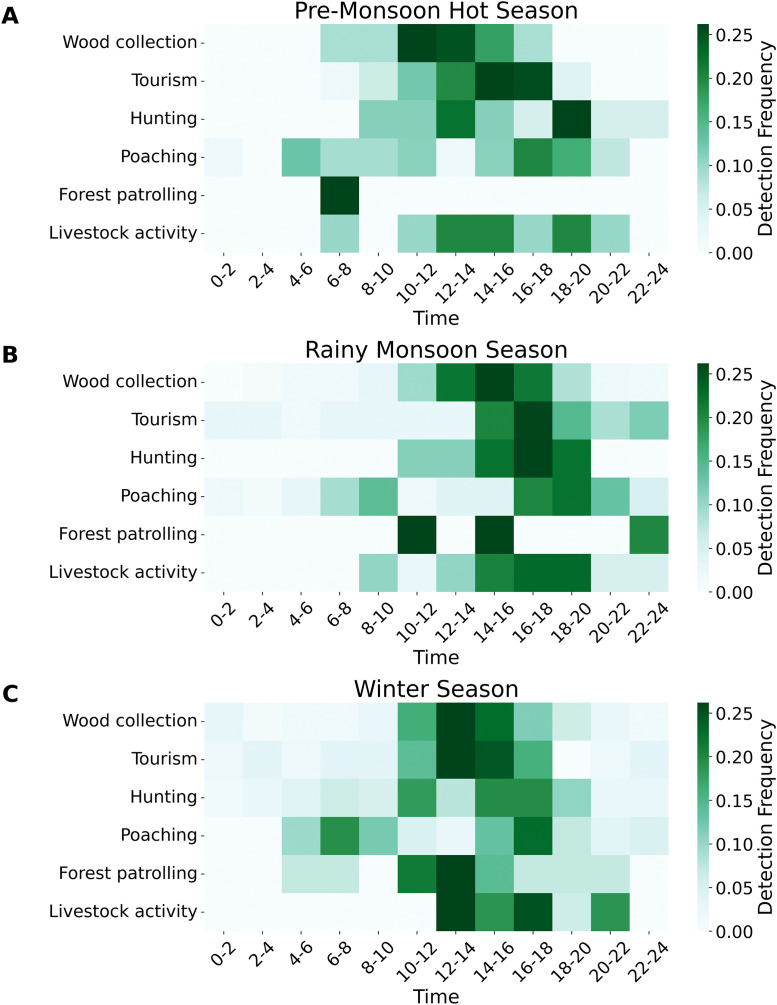
Seasonal and temporal patterns of anthropogenic activities in SNP. **(A)** Pre-Monsoon hot season, **(B)** Rainy monsoon season, and **(C)** Winter Season. The heat map illustrates the frequency and time distribution of various activities, including wood collection, tourism, hunting, poaching, forest patrolling, and livestock activity that distributed across 2-hour intervals over a 24-hour cycle. The intensity of green coloration corresponds to activity frequency, with darker shades indicating higher levels of activity.

The seasonal analysis of anthropogenic intrusions in SNP revealed distinct temporal patterns across the pre-monsoon, rainy monsoon, and winter seasons (Fig 4). Tourism was peaked between 10:00 and 12:00 during the pre-monsoon hot season (Fig 4A), while wood collection was most active between 10:00 and 14:00. The hunting occurred sporadically throughout the day, whereas poaching incidents showed notable peaks between 06:00–08:00 and 14:00–18:00. Livestock activity was peaked in the late afternoon to evening (16:00–22:00), whereas forest patrolling was mostly focused between 6:00 and 8:00. The pattern of activity changed little throughout the rainy monsoon season (Fig 4B). Activities related to wood collection and presence of tourist peaked later in the day, especially between 12:00 and 16:00. While forest patrolling was more active during the 12:00–14:00 hour, hunting and poaching were active between 8:00–12:00 and 14:00–18:00 and peak time for livestock movement was between 16:00 and 18:00. The peak hours for wood collection and presence of tourist were from 12:00–14:00 (Fig 4C). Hunting activity was minimal during the early morning and late evening, with moderate levels observed from mid-morning through the afternoon; poaching events were infrequent and followed a similar temporal pattern. While livestock activity again peaked in the late afternoon (16:00–18:00), forest patrolling was also active in the early morning (6:00–8:00).

**Fig 5:**
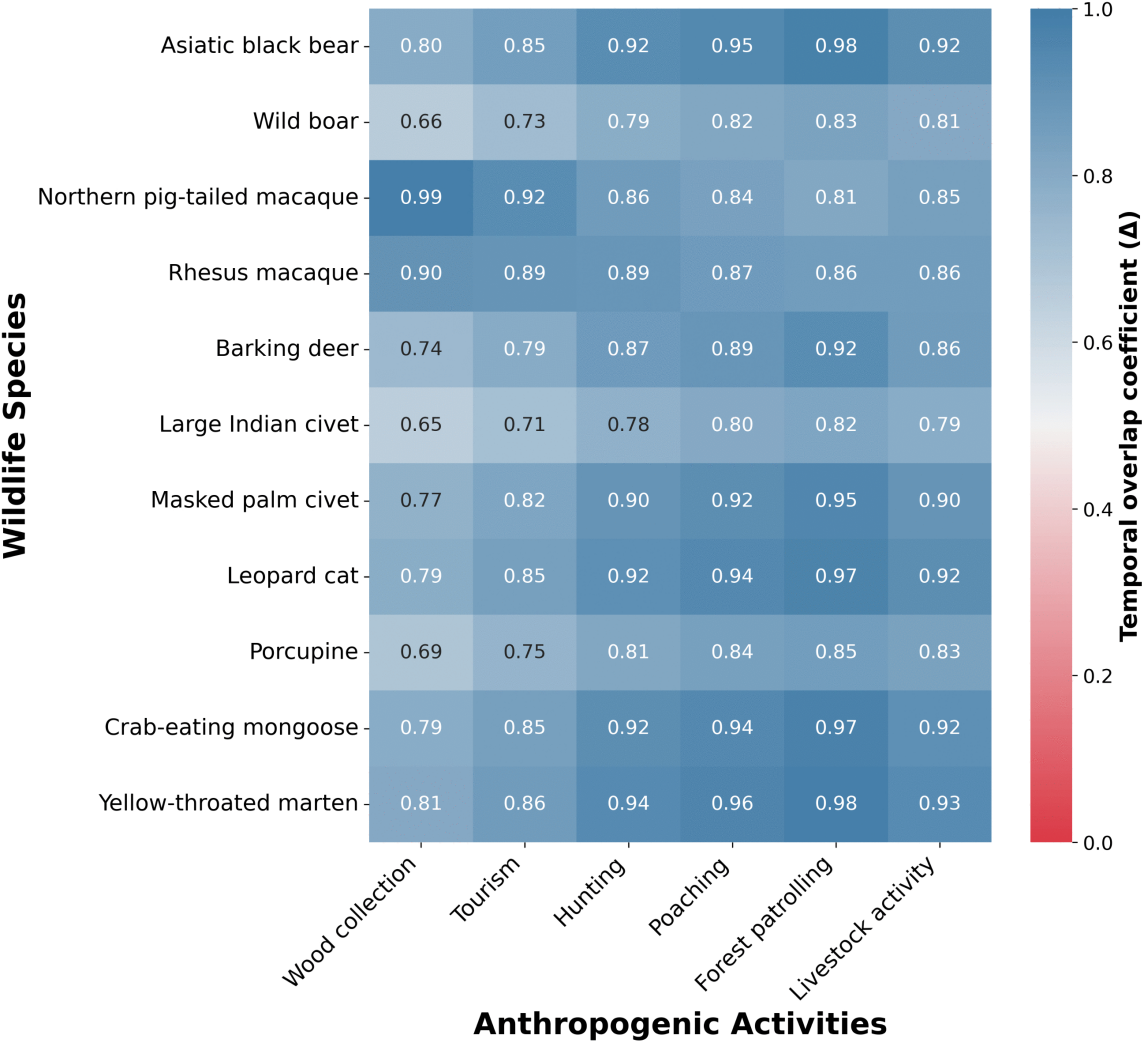
Temporal overlap analysis. Heatmap illustrating the temporal overlap coefficients (Δ) between wildlife species and anthropogenic activities in SNP. Wildlife species are presented on the y-axis and anthropogenic activities including wood collection, tourism, hunting, poaching, forest patrolling, and livestock activity are shown on the x-axis. Color intensity represents the magnitude of temporal overlap (Δ), ranging from low (red) to high (blue), with darker shades indicating stronger temporal co-occurrence between wildlife and human activities.

The temporal overlap analysis demonstrated consistently high coefficients (Δ) between wildlife species and anthropogenic activities within SNP (Fig 5). Corresponding activity patterns (kernel density curves) underlying these overlap estimates are presented in supplementary information (S1 Fig). The result showed that forest patrolling was highest and most consistent overlap across wild species including Asiatic black bear (Δ = 0.98), crab-eating mongoose (Δ = 0.97), leopard cat (Δ = 0.97), and yellow-throated marten (Δ = 0.98). Similarly, high overlap values were observed for hunting (Δ = 0.87–0.94), livestock activity (Δ = 0.86–0.93), and poaching (Δ = 0.80–0.96) across most wild species. In contrast, tourism and wood collection showed comparatively lower, substantial, overlap coefficients. The species-specific variation was evident, with northern pig-tailed macaque showing the highest overlap with wood collection (Δ = 0.99) and tourism (Δ = 0.92), and rhesus macaque also exhibiting strong overlap with these activities (Δ = 0.89–0.90). In contrast, relatively lower overlap values were recorded for large Indian civet and wild boar across most disturbance categories, particularly with wood collection (Δ = 0.65–0.66). We also assessed the correlation analysis between anthropogenic intrusions and wild animal species distribution in SNP of Sylhet that is presented in supplementary information (S2 Fig). The result demonstrated that the northern pig-tailed macaque showed strong positive correlations with wood collection, tourism, hunting, and livestock activity (r = 0.71–0.94, *p* < 0.01–0.001), while the rhesus macaque was positively associated with wood collection and tourism (r = 0.60–0.71, *p* < 0.05–0.01). However, the large Indian civet and masked palm civet showed significant negative correlations with multiple disturbances, including wood collection, hunting, tourism, and livestock activity (r = −0.55 to −0.79, *p* < 0.05–0.01). The barking deer also showed negative associations with wood collection and tourism (r = −0.59, *p* < 0.05). Conversely, the yellow-throated marten showed strong positive correlations with most anthropogenic activities (r = 0.61–0.93, *p* < 0.05–0.001).

## Discussion

PAs play a vital role for conserving wildlife biodiversity, providing secure habitats in which ecosystems can persist with minimal anthropogenic disturbances [43–45]. According to the estimates by the United Nations Development Programme using the Human Development Index, approximately 90% of the world’s poorest populations depend on forests for at least part of their livelihoods [46]. Anthropogenic intrusions significantly alter ecological processes within PAs, influencing wildlife diversity by modifying population dynamics, facilitating the introduction of invasive species, and increasing the risk of local extinction of vulnerable species [47,48]. Previous study has shown that local communities adjacent to PAs encounter two primary challenges of wildlife-related issues: resource degradation and livestock predation along with risks to human safety posed by wildlife moving out from PAs [49]. Other studies have reported that PAs of Achanakmar Tiger Reserve experiences high levels of anthropogenic disturbance, poaching pressure with human settlements and roads constituting the most significant sources of wildlife disturbance [50–52]. Another study reported that local community members collect building poles and fuelwood from the Nimule National Park of Sudan for household needs such as cooking, construction, and charcoal production [9]. These challenges often intensify human–wildlife interactions, negatively affecting local livelihoods and undermining conservation efforts in PAs.

Our study comprehensively evaluated the impact of anthropogenic intrusions on wild animal species within SNP of Sylhet, Bangladesh. By analyzing various anthropogenic intrusions including hunting, poaching, wood collection, tourism, and livestock activity, we determined distinctive and species-specific responses to these disturbances in SNP of Sylhet. Our results provided crucial validation of the occurrence of hunting activities within the SNP of Sylhet. Several video data depicted that hunter was carrying firearms, such as rifles and shotguns, as well as traditional hunting tools like bows and arrows. These visual records not only confirmed the presence of hunters but also highlighted the different hunting methods employed in the region. The presence of such equipment strongly indicates active hunting practices, further corroborating anecdotal reports and community observations. Even when hunting activities do not directly target endangered wild animal species present in the PAs, indiscriminate trapping can severely affect their persistence and ultimately lead to shifts in faunal species composition [18]. Moreover, our study found that hunting activity peaked in the morning and afternoon, likely corresponding to periods of lower human presence and increased wildlife activity. The previous study reported that hunting showed an adverse effects on several species, including large Indian civet, masked palm civet, and barking deer which are frequently primary target due to easy of hunting operation, size, ethno-medicinal value, and market value for meat [53].

Our study demonstrated that, contrary to the expectation of pronounced temporal segregation, overlap coefficients (Δ) were consistently high across wildlife species and anthropogenic activities. This indicates substantial temporal co-occurrence, with many species remaining active during periods that overlap with human use of the forest. However, such overlap should not be interpreted as evidence of tolerance or adaptation, as camera trap–based detections cannot resolve underlying behavioral mechanisms, including habituation, fine-scale spatial displacement, or variation in detection probability across disturbance contexts. Notably, hunting activities showed relatively high temporal overlap with many wild animals in SNP of Sylhet. While illegal hunting has been widely identified as a major driver of wildlife population declines, particularly in developing countries [9,53], the present results represent temporal co-occurrence patterns and do not provide direct evidence of ecological impacts. Therefore, these findings should be interpreted cautiously, as high overlap does not necessarily indicate increased vulnerability or direct negative effects without additional behavioral or population-level evidence.

Previous study reported that poaching is often driven by financial gain through the trafficking of wild animals or their body parts [54]. In line with the present findings, a study by Shazali et al. [9] reported that poaching and encroachment into the PAs limited the large mammal’s biodiversity in Nimule National Park, South Sudan. Several investigations have shown that hunting, poaching, and wood collection within PAs as substantial hazards to wildlife distribution and their natural habitats [47,53,55,56]. In these regions, limited livelihood options often lead local communities to exploit PAs [57], while underfunded conservation efforts and weak law enforcement further exacerbate illegal activities such as hunting, human encroachment [58]. Consistent with the present study, human encroachment alters primate habitats and drives spatiotemporal shifts, resulting in the use of forest–agricultural zones for feeding and foraging, highlighting their reliance on both natural and anthropogenic landscapes [59].

Our study highlighted that recreational tourism was a prominent anthropogenic intrusion in SNP of Sylhet. The temporal analysis revealed that tourism has high overlap with wild animal species such as northern pig-tailed macaque and rhesus macaque, deviating from the predominantly diurnal patterns. Primates are often highly adaptable and may show a tendency to associate with human presence, particularly in areas where food resources are available from human. As a result, this behavioral flexibility can promote increased activity during periods when human-derived resources are accessible. Additionally, the relatively low perceived risk from non-lethal disturbances from tourists, may reduce the need for strict temporal avoidance. Ecotourism can generate revenue to support conservation initiatives, strengthen local economies, and enhance biodiversity awareness [60], our findings suggest that unmanaged or intensive tourism activities may also influence wildlife behavior and activity patterns within PAs. Study reported that recreational visits to PAs provide opportunities for public enjoyment and enhance conservation awareness; however, they can also exert significant impacts on wildlife biodiversity and conservation outcomes [61]. In this context, unregulated tourism in SNP, Sylhet, may disrupt wildlife behavior, alter habitat use, and exacerbate human–wildlife conflicts.

This study offers multiple key insights that may guide the planning and implementation of future conservation strategies in SNP of Sylhet. First, to prevent hunting, poaching, and wood collection in SNP, it is essential to establish stringent anti-poaching legislation and strict filed application with heavy penalties. Second, facilitating the frequency of patrolling and monitoring using digital technology such as drones and camera traps throughout the entire year. Third, the installation of checkpoints at strategic entry and exit routes around the SNP could significantly restrict unlawful access. Fourth, restricting tourism into selected areas and ensuring that visitors remain within designated zones that can substantially reduce the adverse impacts of recreational activities on wildlife. Fifth, active involvement of local communities in conservation initiatives could be started integrated with the promotion of environmentally sustainable alternative livelihoods including sustainable agriculture, handicrafts, and ecotourism. Finally, the priority should be given to school-based education programs that could enhance the long-term consequences and awareness regarding ecology, biodiversity, and wildlife.

Although this study provides valuable insights into anthropogenic activities within the SNP, it has several limitations. First, several camera traps were either broken or stolen by hunters and poachers. However, we promptly replaced those cameras to ensure uninterrupted monitoring. Second, huge rainfall and flood during the camera trapping aslo presented challenges to field operations. Third, the study recorded anthropogenic intrusions within SNP through camera trapping but did not analyze the socio-economic determinants influencing these behaviors. Addressing these limitations in future studies would improve conservation effectiveness in SNP as well as other PAs of Bangladesh.

## Conclusions

This study provides vital insights into the anthropogenic intrusions in SNP of Sylhet, Bangladesh. Importantly, the study identified several anthropogenic activities including hunting, poaching, wood collection, livestock activity, and extensive tourism in SNP, highlight the increasing anthropogenic pressures threatening this biodiversity hotspot. These anthropogenic disturbances influence wildlife activity patterns, with many species exhibiting shifts in temporal activity and spatiotemporal dynamics in response to human presence within the study area. Our study demonstrated that the interaction of anthropogenic intrusions and wild animal species activity underscores the pressing necessity for sustainable wildlife management and conservation techniques in SNP of Sylhet. The study highlights the need to strengthen conservation strategies, enforce stricter anti-poaching measures, promote responsible eco-tourism, and actively involve local communities in forest stewardship, while improved monitoring can further support the conservation of SNP’s unique biodiversity. To the best of our knowledge, this is the first study of camera trap monitoring to investigate anthropogenic intrusions in SNP of Sylhet, Bangladesh.

## Supporting information

S1 Fig Species-specific temporal activity patterns in relation to anthropogenic activities. Species-specific temporal activity patterns relative to individual anthropogenic activities, with each panel (A–F) representing a different activity and multiple species shown as separate subplots within each panel. Panel A: Wood collection; B: Tourism; C: Hunting; D: Poaching; E: Forest patrolling; F: Livestock activity. Each subplot illustrates the diel activity pattern of a single wildlife species in relation to the corresponding anthropogenic activity.
(PDF)

S2 Fig Correlation heatmap. Correlation heatmap illustrated the relationships between wild animal species and various anthropogenic activities in SNP. Wild animal species are shown on the y-axis, whereas various anthropogenic activities such as wood collection, tourism, hunting, poaching, forest patrolling, and livestock activity are depicted on the x-axis. The color scale denotes correlation coefficients, extending from positive (blue) to negative (red), with darker hues signifying stronger correlations. Notable associations are shown by asterisks: *p < 0.05, **p < 0.01, ***p < 0.001.
(PDF)
