# Supplementary figures and images for: Camera traps reveal extensive anthropogenic impacts inside protected areas in Bangladesh
Source: PLoS One. 2026 Apr 28;21(4):e0347792. doi: 10.1371/journal.pone.0347792 (PMC13123977; doi:10.1371/journal.pone.0347792)

A)

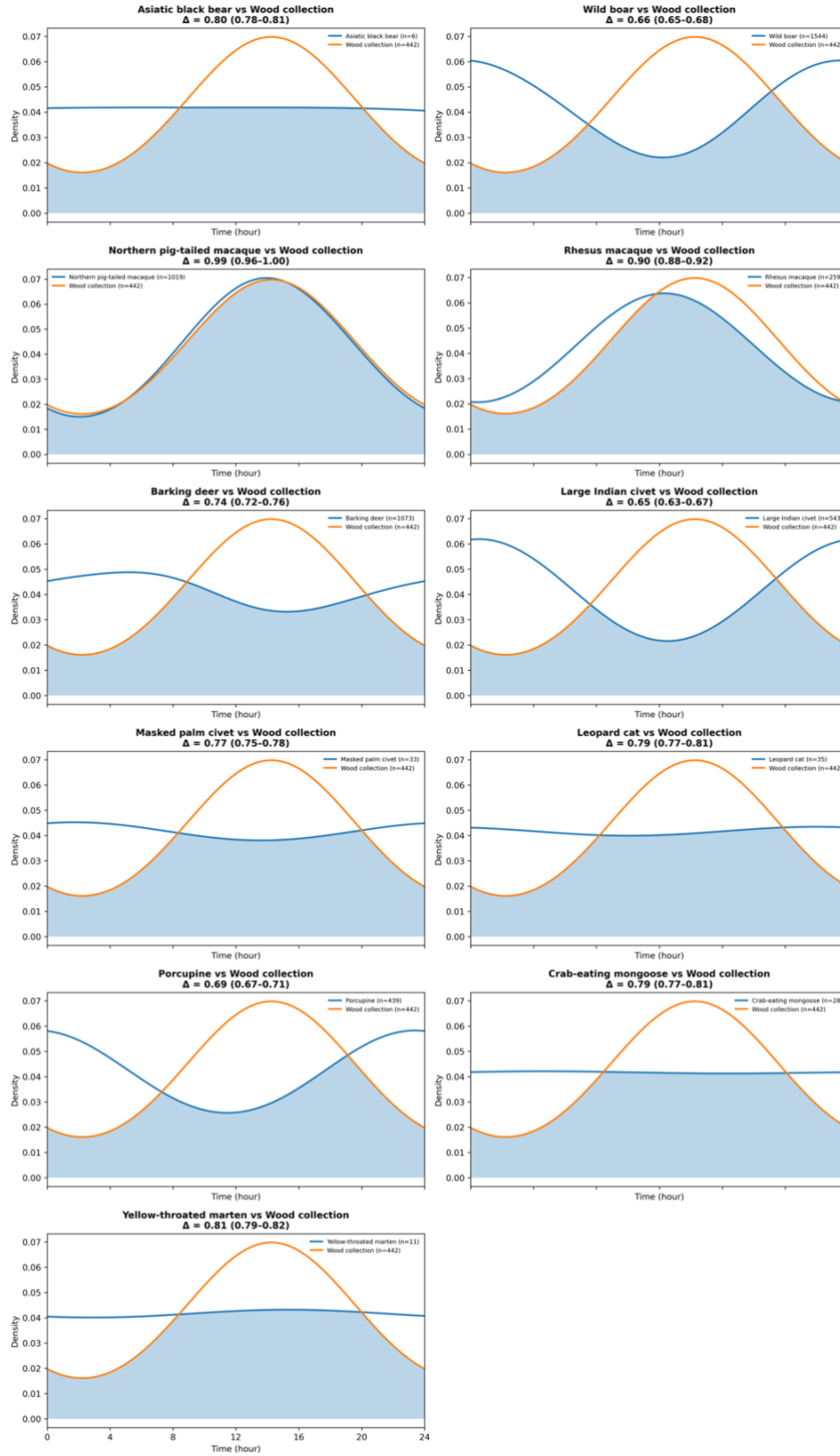

B)

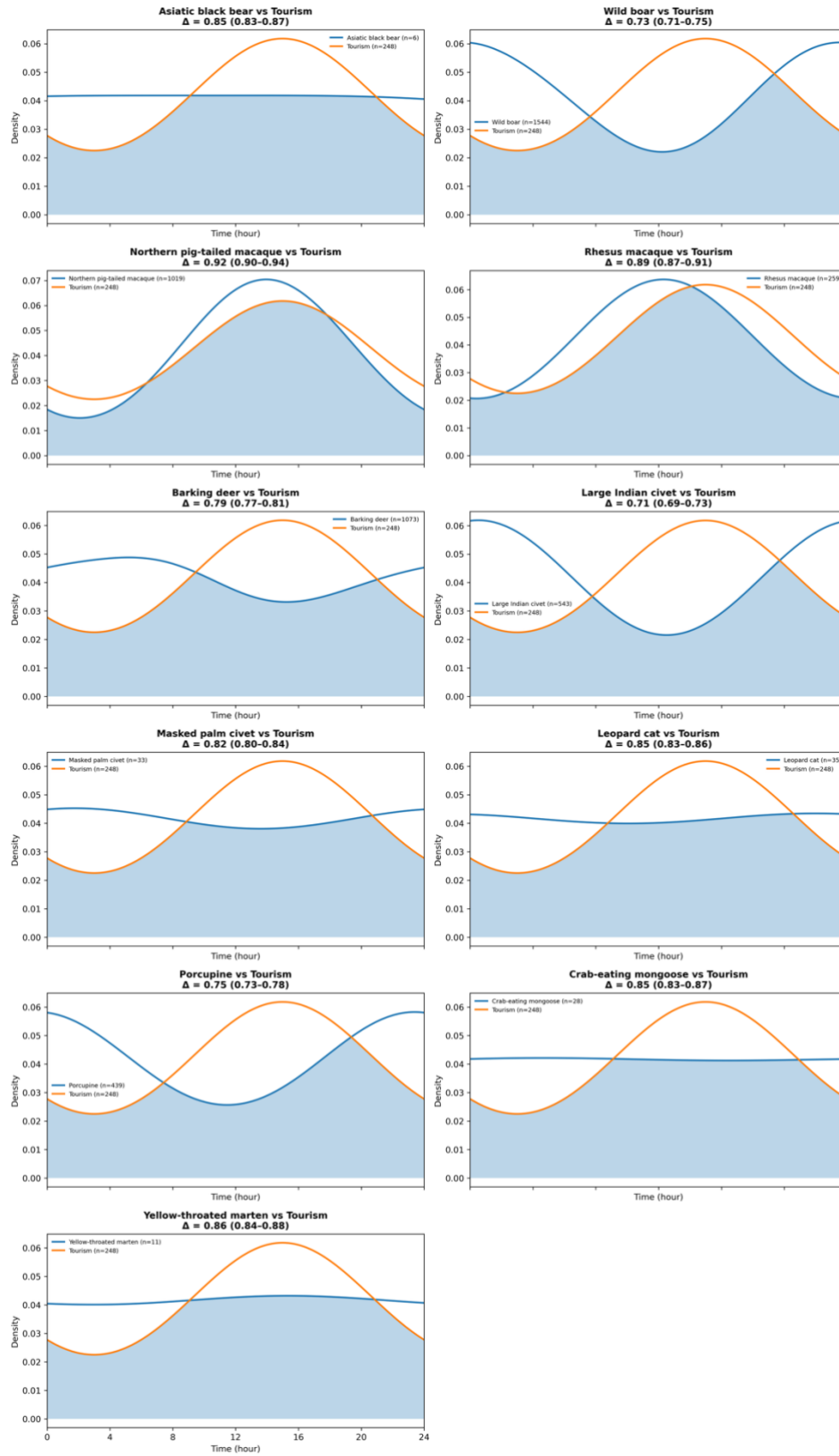

C)

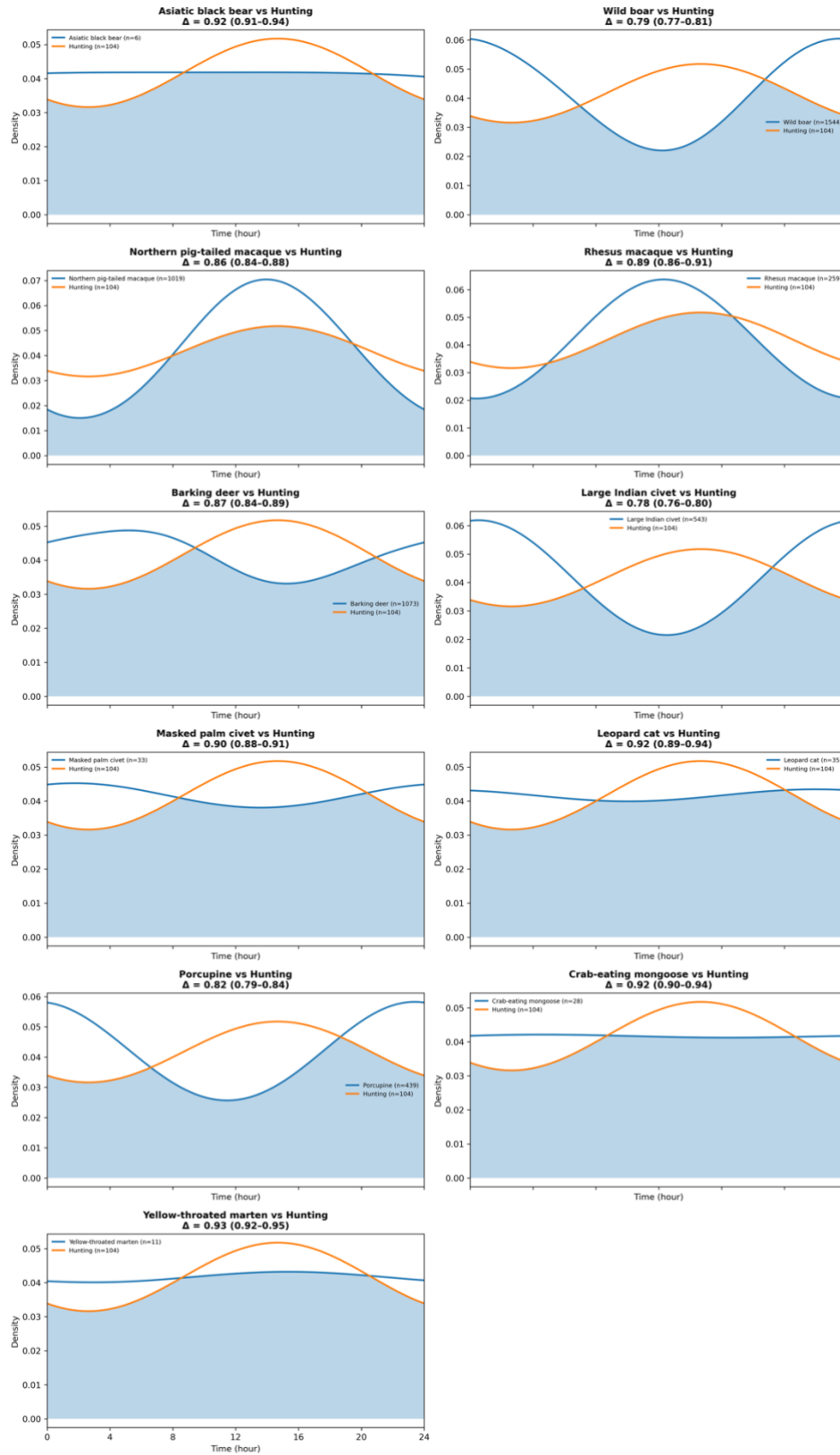

D)

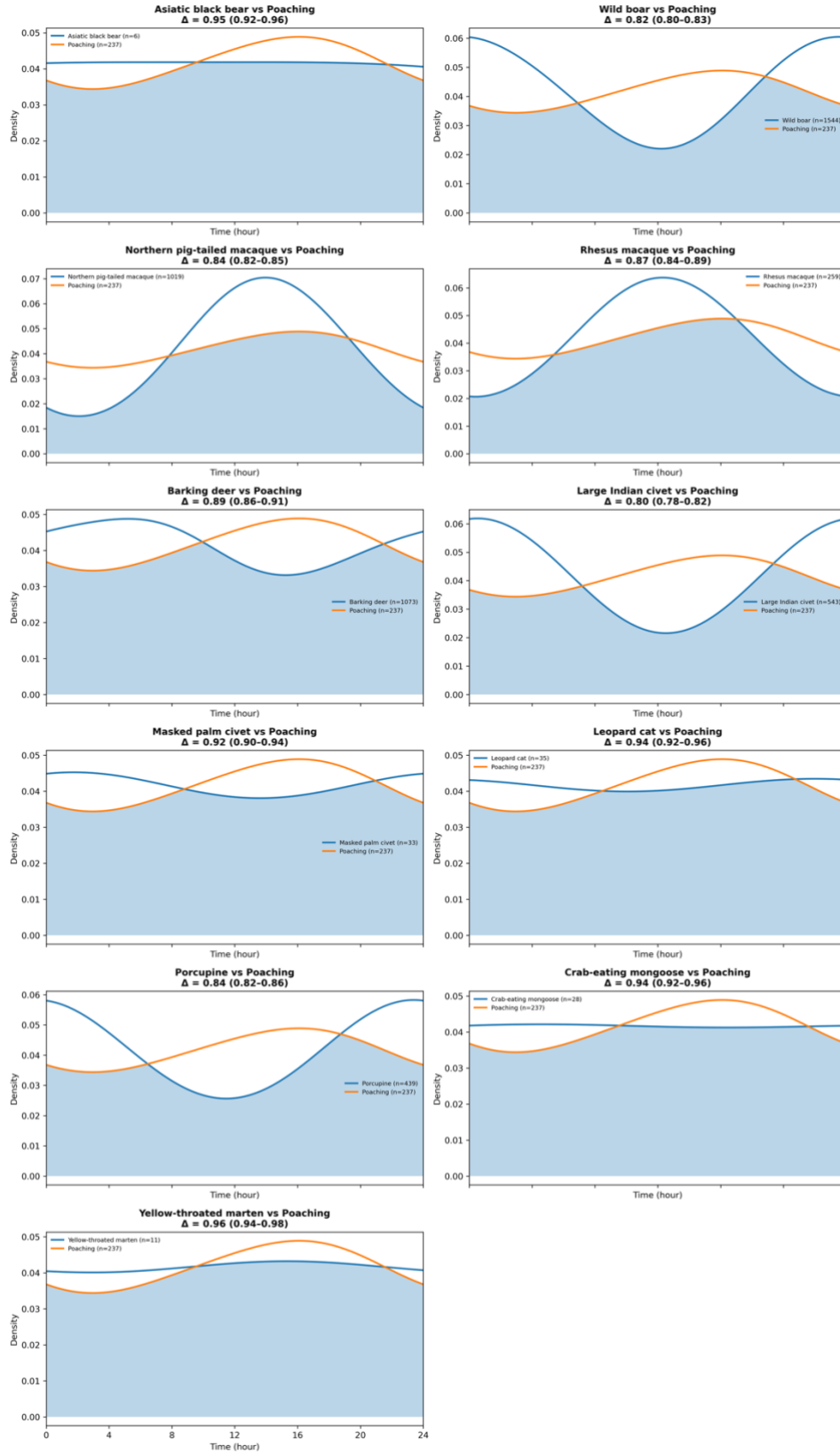

E

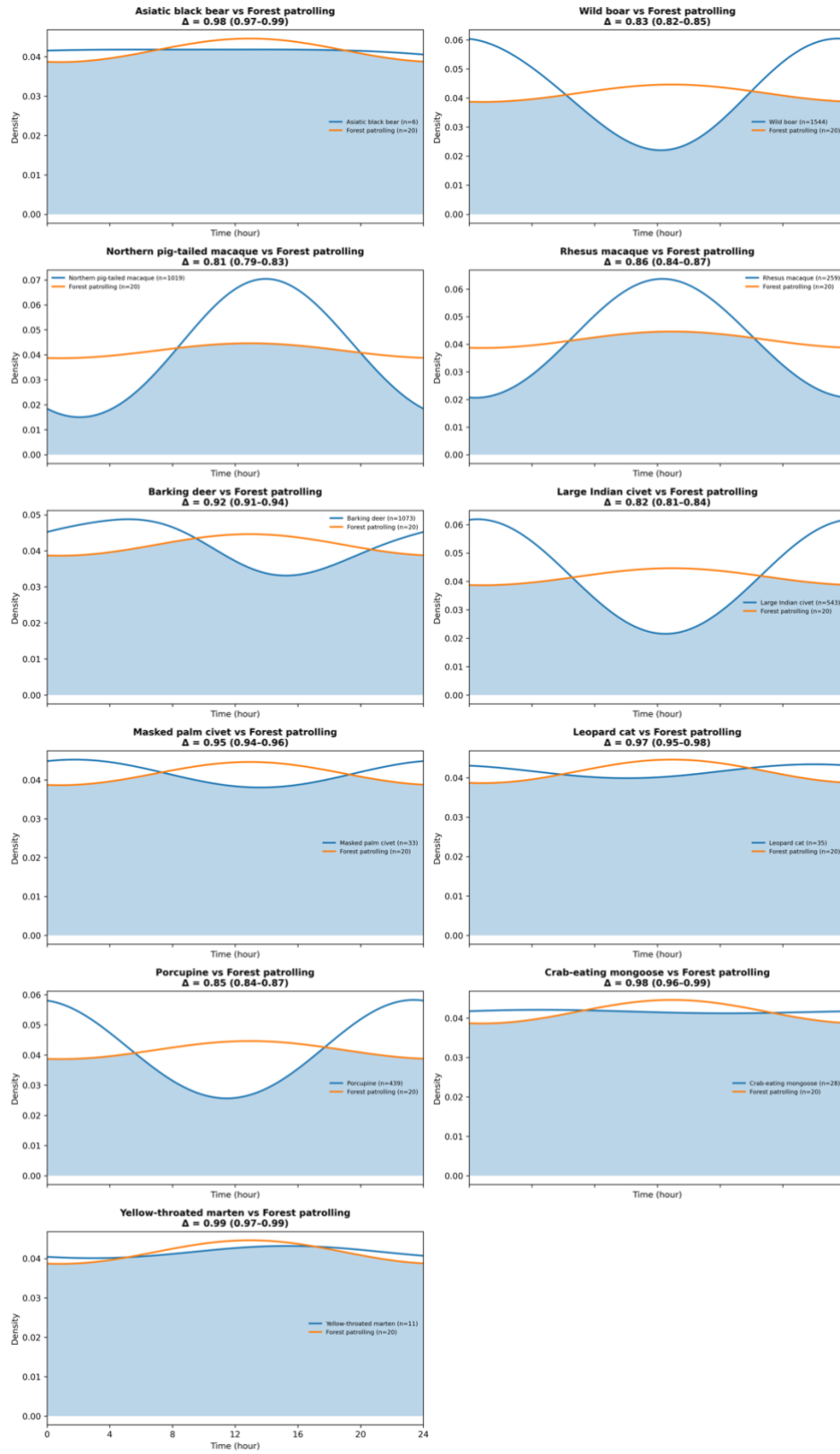

F)

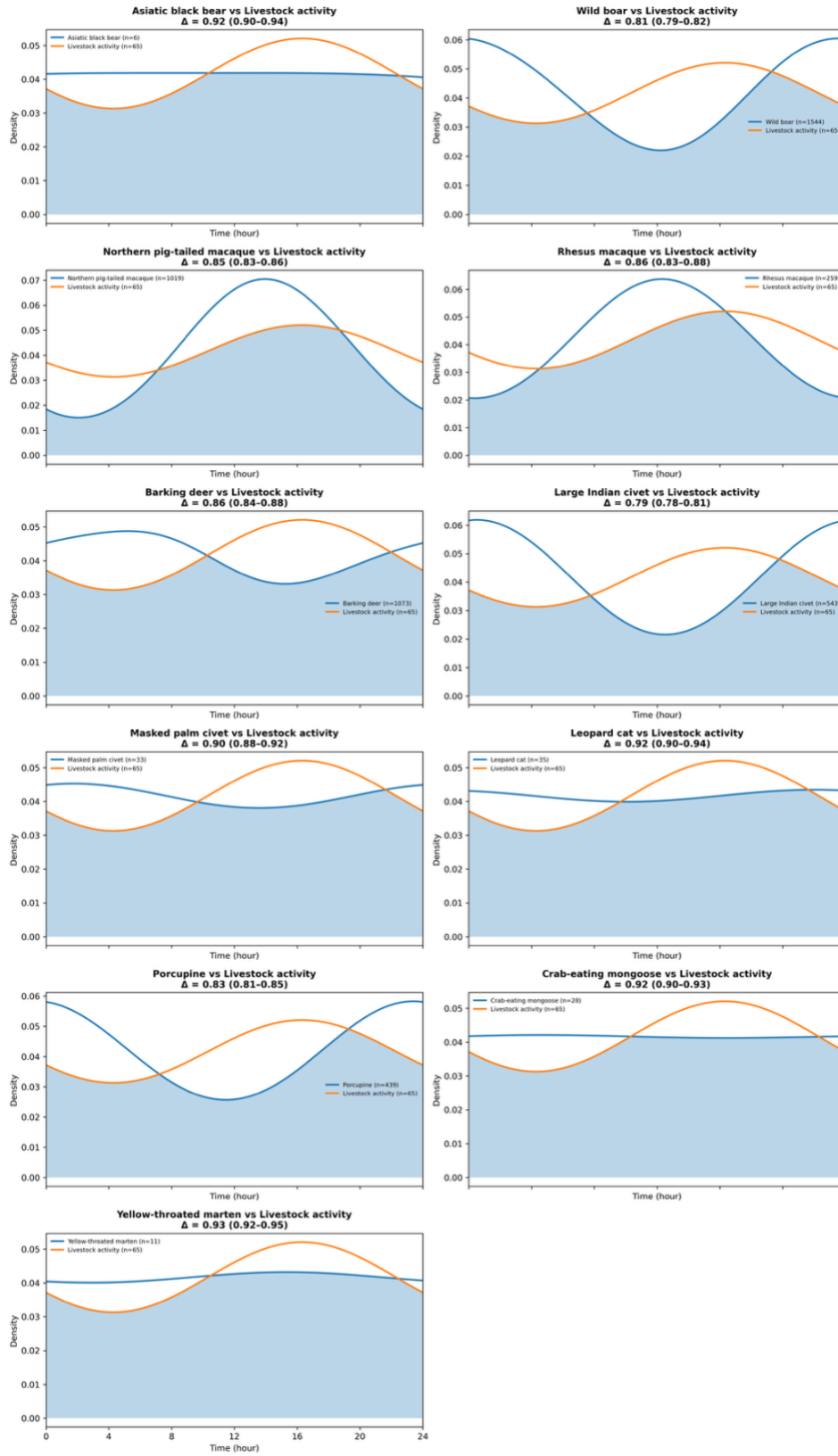

Supplement: S1 Fig — Species-specific temporal activity patterns relative to individual anthropogenic activities, with each panel (A–F) representing a different activity and multiple species shown as separate subplots within each panel. Panel A: Wood collection; B: Tourism; C: Hunting; D: Poaching; E: Forest patrolling; F: Livestock activity. Each subplot illustrates the diel activity pattern of a single wildlife species in relation to the corresponding anthropogenic activity. (PDF) [file pone.0347792.s001.pdf]

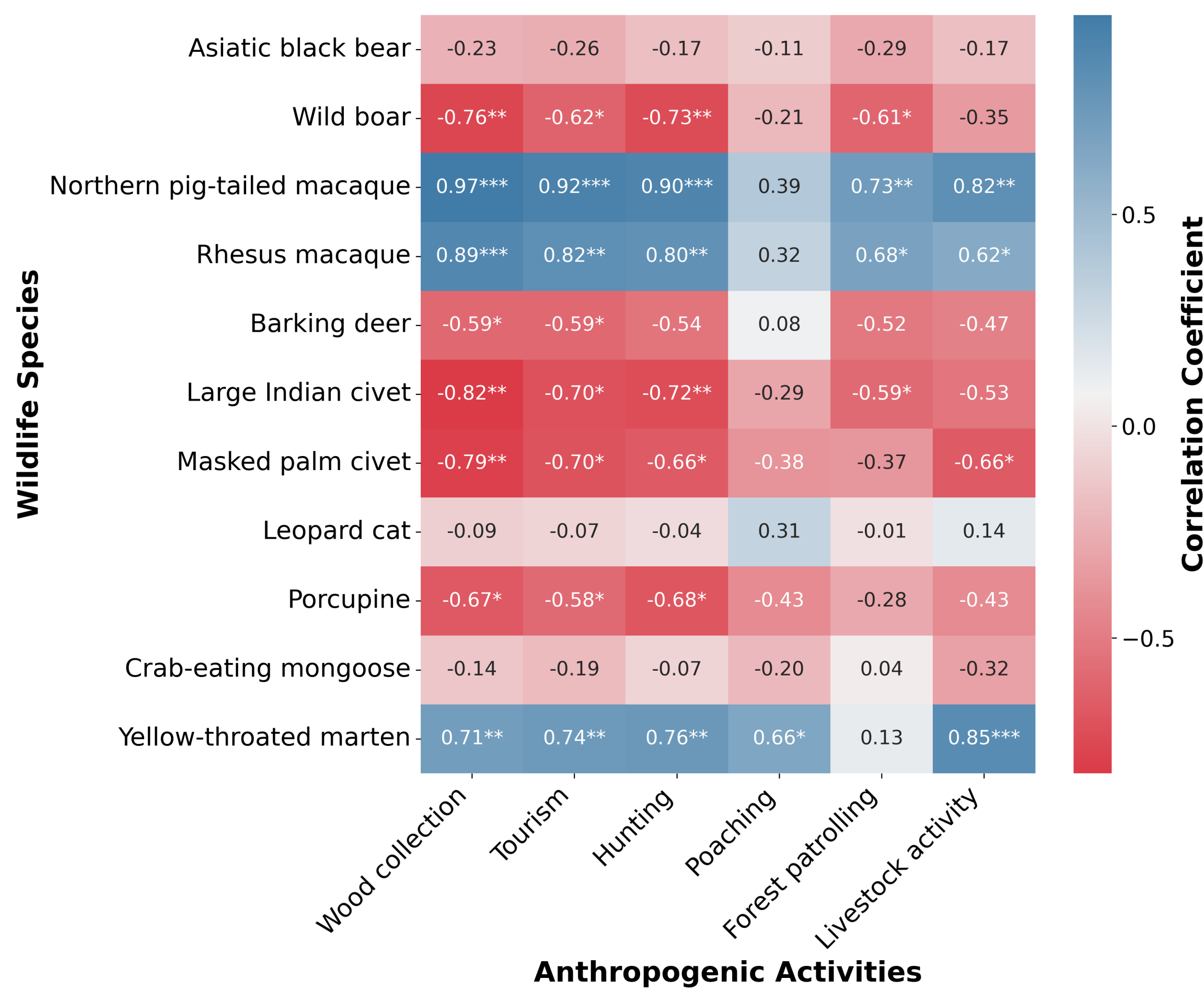

Supplement: S2 Fig — Correlation heatmap illustrated the relationships between wild animal species and various anthropogenic activities in SNP. Wild animal species are shown on the y-axis, whereas various anthropogenic activities such as wood collection, tourism, hunting, poaching, forest patrolling, and livestock activity are depicted on the x-axis. The color scale denotes correlation coefficients, extending from positive (blue) to negative (red), with darker hues signifying stronger correlations. Notable associations are shown by asterisks: *p < 0.05, **p < 0.01, ***p < 0.001. (PDF) [file pone.0347792.s002.pdf]
